# Conventional and food‐to‐food fortification: An appraisal of past practices and lessons learned

**DOI:** 10.1002/fsn3.1133

**Published:** 2019-08-05

**Authors:** Flora Josiane Chadare, Rodrigue Idohou, Eunice Nago, Marius Affonfere, Julienne Agossadou, Toyi Kévin Fassinou, Christel Kénou, Sewanou Honfo, Paulin Azokpota, Anita R. Linnemann, Djidjoho J. Hounhouigan

**Affiliations:** ^1^ Faculty of Agronomic Sciences, Laboratory of Food Science University of Abomey‐Calavi (LSA/FSA/UAC) Abomey‐Calavi Benin; ^2^ Ecole des Sciences et Techniques de Conservation et de Transformation des Produits Agricoles Université Nationale d'Agriculture (ESTCTPA/UNA) Sakete Benin; ^3^ Faculté des Sciences Agronomiques, Laboratoire de Biomathématiques et d'Estimations Forestières Université d'Abomey‐Calavi (LABEF/FSA/UAC) Abomey‐Calavi Benin; ^4^ Ecole de Gestion et de Production Végétale et Semencière Université Nationale d'Agriculture (EGPVS/UNA) Ketou Benin; ^5^ Ecole de Nutrition et des Sciences et Technologies Alimentaires, Faculté des Sciences Agronomiques Université d'Abomey‐Calavi (ENSTA/FSA/UAC) Abomey‐Calavi Benin; ^6^ Food Quality and Design (FQD/WUR) Wageningen University and Research Wageningen The Netherlands

**Keywords:** food fortification, malnutrition, micronutrient deficiencies, outcomes

## Abstract

Food fortification is an important nutrition intervention to fight micronutrient deficiencies and to reduce their incidence in many low‐ and middle‐income countries. Food fortification approaches experienced a significant rise in the recent years and have generated a lot of criticism. The present review aimed to shed light on the actual effect of food fortification approaches on the reduction of malnutrition. A set of 100 articles and reports, which have dealt with the impact of food fortification on malnutrition, were included in this review. This review identified a broad selection of local raw materials suitable for a food‐to‐food fortification approach.

## 1 INTRODUCTION

Micronutrient deficiencies often cause malnutrition that is a crucial public health problem, especially in developing countries (Ramakrishnan, Goldenberg, & Allen, 2011). Indeed, they generate several diseases either infectious or chronic and therefore impacts the life's quality and epidemiological parameters such as morbidity and mortality (Verma, 2015). As a consequence, this type of malnutrition leads to premature death, disability, and reduced work capacity (Black et al., 2013) and more often reaches children and women of reproductive age (Method & Tulchinsky, 2015). Food fortification is considered as the most appropriate preventive approach against malnutrition caused by micronutrient deficiencies (Bhagwat, Gulati, Sachdeva, & Sankar, 2014). For many years, food fortification has been used as a cost‐effective means to prevent micronutrient malnutrition (Method & Tulchinsky, 2015). Considerable studies have been carried out to develop food fortification in developing countries (Akhtar, Anjum, & Anjum, 2010; Bhagwat et al., 2014; Mishra, 2011). However, effectiveness of food fortification approaches to improve nutritional status has to be coherently analyzed and evidenced. In order to evaluate the most important global trends and historical patterns in food fortification, large databases are required to study the different types of fortification. Indeed, data relevant to the history, impacts, and challenges of food fortification are scattered across literature and existing reviews concern a few countries. Therefore, though information on food fortification successes and failures may be difficult to assess and compare, key factors of success or failure of interventions need to be identified to inform policymakers and assist countries in the design and implementation of appropriate fortification programs. The present review: (a) presents the history of knowledge and know‐how from conventional food fortification to food‐to‐food fortification,^1^ (b) assesses challenges of food fortification, and (c) documents best practices and benefits of food‐to‐food fortification approaches.

## 2 METHODOLOGY

A comprehensive literature search was conducted using Web of Science/Knowledge, Google Scholar (http://scholar.google.com), Elsevier ScienceDirect (http://www.sciencedirect.com), and Springer Online Journals (http://link.springer.com). The search syntax contained the following keywords: food fortification, micronutrient deficiencies, fortified food, food fortification impact, and food‐to‐food fortification. The focus was on peer‐reviewed articles and government reports between 1990 and 2016. In addition, a few earlier milestone articles published before 1990 were included. Other major relevant syntheses were also reviewed including those by the World Health Organization (WHO) and the International Food Policy Research Institute (IFPRI). Initially, a total of 410 publications were identified. Further review of the title, abstract, and full texts of these documents led to the elimination of 250 citations due to their lack of relevance. Finally, 160 articles and reports were used of which 100 were actually included in this review.

## 3 RESULTS

### 3.1 Prevalence of undernutrition and micronutrient deficiencies in the world

Malnutrition (overnutrition, undernutrition, and micronutrient deficiencies) is a physiological state characterized by a low or high quantity of macronutrients, micronutrients, or both in human's organism (Ortiz‐Andrellucchi, Ngo, & Serra‐Majem, 2016). Currently, several cases of obesity and overweight due to overnutrition are recorded worldwide (IFPRI, 2016). Meanwhile, undernutrition and micronutrient deficiencies are recurrent and have significant negative effects on public health (Lopez, Mathers, Ezzati, Jamison, & Murray, 2006). The prevalence of undernutrition varies considerably according to countries. In developing countries, rural people are the most subjected to undernutrition (Shetty, 2009). Indeed, micronutrient deficiencies are often associated with low income and poor access to nutritious foods, situations that are frequent in rural areas (Shetty, 2009). According to recent estimations, about two billion people suffer from micronutrient deficiencies (Allen, de Benoist, Dary, & Hurrell, 2006). Micronutrient deficiencies account for about 7.3% of the global burden of disease, with iron and vitamin A deficiencies among the 15 leading causes of the global disease burden (WHO, 2000). Animal foods are important sources of protein and of micronutrients such as iron, zinc, vitamin A, and vitamin B_12_. Unfortunately, in developing countries most people cannot afford these foods in their daily diet. As a result, they suffer from micronutrient deficiencies. Folic acid, vitamin D, selenium, and zinc deficiencies, although less recognized, are important as well. A lack of those micronutrients represents a major threat to the health and development of populations in particular in developing countries (Bain et al., 2013; Müller & Krawinkel, 2005).

Many children worldwide suffer from nutritional deficiencies, which can negatively affect their physical and mental development and increase susceptibility to infections. Moreover, undernutrition amplifies the effect of every disease, including measles and malaria. Undernutrition (53%) causes as much mortality of children younger than 5 years as diarrhea (61%), malaria (57%), pneumonia (52%), and measles (45%; Black, Morris, & Bryce, 2003; Bryce, Boschi‐Pinto, Shibuya, & Black, 2005). In addition, according to Black et al. (2008), women and children are the major targets suffering from consequences of micronutrient deficiency such as poor pregnancy outcomes, children's impaired mental, and physical development. Up to 3.1–3.5 million of children under 5 years old die every year and women of reproductive age living in low‐ and middle‐income countries because of undernutrition (fetal growth restriction, suboptimum breastfeeding, stunting, wasting, and deficiencies of vitamin A, iodine, zinc, iron, vitamin D deficiency, rickets, osteomalacia, and thyroid deficiency) (Black et al., 2008, 2003; Mandelbaum, 2004; Method & Tulchinsky, 2015). Zinc deficiency is a risk factor with adverse long‐term effects on growth, immunity, and metabolic status of surviving offspring (Harika et al., 2017). Therefore, elimination of these deficiencies is essential, not only to improve health, but also for sustained economic growth and national development (Mishra, 2011).

### 3.2 Classical food fortification: definition and importance

The nutrient intake of basic foods, seasonings, or condiments may be enhanced through a fortification that increases the content of essential micronutrients, such as vitamins and minerals (Mannar & Gallego, 2002). One way to fortify foods is to incorporate synthetic micronutrients to it (Zimmermann, Muthayya, Moretti, Kurpad, & Hurrell, 2006). In many developing countries, the most widely used vehicles for fortification are among the most commonly consumed foods, including oils and fats, milk, sugar, salt, rice, wheat, or maize flour. Some factors related to food fortification such as level of fortification; bioavailability of fortificants; and amount of fortified food consumed have a significant effect on health (Verma, 2015; see Das, Salam, Kumar, & Bhutta, 2013 for more information). Classical food fortification with zinc is very common; in case, the vehicle is cereal flour at a recommended level (100 mg zinc/kg for wheat flour; Brown, Hambidge, Ranum, & Zinc, 2010) of zinc fortification, of which a lower level may have no significant effect on the nutrient improvement of the cereal (Brown, Peerson, Baker, & Sonja, 2009).

Food fortification leads to rapid improvement in the micronutrient status of a population, and at a reasonable cost, especially if advantage is taken from existing technology and local distribution networks. Rice fortification has an advantage to benefit to almost half of the world's population (>3 billion people consumed rice as their main staple worldwide; de Pee, Tsang, Zimmerman, & Montgomery, 2018). Thus, rice can be considered as one of the best staple food vehicles for food fortification in developing counties regarding a population‐level intervention (Moench‐Pfanner, Laillou, & Berger, 2012). Fortification of rice flour with iron, zinc, and folate allows children under 5 years old, having a rapid iron and zinc absorption, to improve their growth and micronutrient status (Hettiarachchi, Hillmers, Liyanage, & Abrams, 2004). Fortifying flour is much simpler because the nutrients that are available in powdered form can successfully be mixed into the flour. As such, rice flour was recommended as a suitable vehicle for fortification.

Long‐term measures have been implemented to combat vitamin A and iron deficiencies, in particular, fortification of cotton oil with vitamin A and of wheat flour with iron, zinc, folic acid, and vitamin B. Multiple micronutrient fortification appears relatively more beneficial and should be considered because multiple micronutrient deficiencies coexist in many cases (Table 1). This consideration justifies why many fortification programs are oriented toward multi‐micronutrients and vehicles chosen adequately for a good acceptability of fortified foods by the target group. Food fortification can take several forms, and different techniques and procedures can be used (Liyanage & Hettiarachchi, 2011).

**Table 1:** Examples and outcomes of classical food fortification

Fortified food	Improved nutrient(s)	Consumption of the fortified foods	Subjects	Outcomes	Limitations	References
Food fortification with a micronutrient						
Fish sauce	Iron	Consumption of 10 ml per day of a sauce that was fortified with 100 mg of iron (as NaFeEDTA) per 100 ml	The subjects were nonpregnant anemic female factory workers in Vietnam	It significantly reduces iron deficiency and iron‐deficiency anemia after 6 months in the group receiving the fortified sauce compared to the placebo control group		FAO and OMS (2006)
Rice meal	Iron	Iron‐fortified rice meal (15 mg of iron per day as ferric pyrophosphate)	Young children (5–9 years)	The prevalence of iron deficiency was significantly reduced There was a significant decrease in median blood lead concentration The prevalence of blood lead levels 10 g/dl was significantly reduced.	The study was of short duration (16 weeks) and blood lead was only measured twice	Zimmermann et al. (2006)
Wheat flour and maize meal	Iron	The iron compound (sodium iron ethylenediaminetetraacetate[NaFeEDTA], ferrous fumarate, or ferrous sulfate) was varied and dosed at rates according to the WHO guidelines for consumption of 75–149 g/day of wheat flour and >300 g/day of maize meal and tested again for 150–300 g/day for both	Three countries were selected for the trials: Kenya, South Africa, and Tanzania	The levels of iron compounds used, in accordance with the WHO guidelines, do not lead to changes in the baking and cooking properties of the wheat flour and maize meal	This trial has not covered all the possible dosage levels of the WHO guidelines, nor by any means all of the possible end‐use products of wheat flour and maize meal	Randall, Johnson, & Verster (2012)
Soy sauce	Iron (as NaFeEDTA)	Daily consumption of 5 mg or 20 mg iron in the fortified sauce	Children	Very effective in the treatment of iron‐deficiency anemia in children; positive effects were seen within 3 months of the start of the intervention	—	FAO and OMS (2006)
Rice	Iron	Rice is fortified at a level likely to lead to approximately equal supplemental iron absorption in both groups. A 10‐kg child in the control group would receive ~10 mg Fe/d (a dose of 20 drops of iron solution thrice weekly)	Infants and young children (6–24 months old)	Fortifying rice with iron may improve iron status at least as well as providing free iron drops	—	Beinner, Velasquez‐Melendez, Pessoa & Greiner (2010)
Porridge cereals	Zinc	A 30 g dry weight of an iron‐fortified cereal porridge and a separate dose of an aqueous multivitamin (MV) supplement between meals (control group), the same porridge and MV with 3 mg Zn added to the supplement dose (ZnSuppl group), or the porridge with added zinc (150 mg/kg dry weight) and MV without zinc (ZnFort group)	6‐ to 8‐month‐old Peruvian children	Increase linear growth and weight gain by a small, but highly significant, amount	A fortified porridge did not significantly affect the children's physical growth	Brown et al. (2009)
Cereals flour	Zinc	The recommended maximum level of zinc fortification is 100 mg zinc/kg wheat flour (100 ppm)	Young children and pregnant and lactating women and adult men	Zinc fortification of cereal flour is a safe and appropriate strategy for enhancing the zinc status of population subgroups who consume adequate amounts of fortified cereal flour	Greater levels of fortification may adversely affect the sensory properties of food items prepared with such flour. Fermentation of flour reduces the level of zinc fortification that is required to meet the theoretical needs for absorbed zinc.	Brown et al. (2010)
Margarine	Vitamin A	Consumption of 27 g of vitamin A‐fortified margarine per day for a period of 6 month	Preschool‐aged children	Reduction in the prevalence of low serum retinol concentrations from 26% to 10%		FAO and OMS (2006)
Wheat flour bun (pandesal)	Vitamin A	A 60‐g vitamin A‐fortified pandesal was consumed by the children 5 day/week for 30 weeks	Children aged 6–13 years attending for rural schools in the Philippines	Vitamin A fortification modified significantly serum retinol effect Daily consumption of vitamin A‐fortified pandesal improved the vitamin A status of Filipino school‐age children with marginal‐to‐low initial serum retinol concentrations.	—	Solon et al. (2000)
Milk	Vitamin D	A 710 ml of vitamin D‐fortified (total 300 IU or 7.5 µg) milk daily	Mongolian school‐age children (22 girls and 24 boys) aged 9–11 years	After one month of drinking milk, all children had an increase in height and weight	—	Ganmaa et al. (2008)
Multiple micronutrient food fortification						
Dairy products such as natural low‐fat cheese and lactose‐reduced yogurt	Calcium and vitamin D	Consumption of calcium (1,000 mg) and 200 IU (5 µg) vitamin D daily during 2 years	Girls (10−12 years)	Increasing calcium intake by consuming cheese appears to be more beneficial for cortical bone mass accrual than the consumption of tablets containing a similar amount of calcium.		Cheng et al. (2005)
Maize grain	Iron and vitamin A	Maize grain was milled and fortified in two custom‐designed mills installed at a central location in the camp, and a daily ration of 400 g per person was distributed twice monthly to households as part of the routine food aid ration. Micronutrient fortificant added to 1 kg of maize meal (Vitamin A [mg RE] 2,100 Thiamin (mg) 4.4 Riboflavin (mg) 2.6 Nicotinamide (mg) 35.0 Vitamin B 6 (mg) 2.5 Vitamin B 12 (mg) 10.0 Folic acid (mg) 1.5 Fe (mg) 35.0 Zn (mg) 20.0	Adolescents (10–19 years), children (6–59 months) and women (20–49 years)	During the intervention period, mean Hb increased in children and adolescents Anemia decreased in children by 23.4% Serum transferring receptor indicating an improvement in the Fe status of adolescents In adolescents, serum retinol increased and vitamin A deficiency decreased by 26.1%	Hb did not increase in women Anemia was not significant change in adolescents or women.	Seal et al. (2007)
Staples, condiment, and processed foods	Single, dual, or multiple micronutrients (iron, folic acid, zinc, vitamin A, iodine, vitamin D, and calcium)	—	Children, adolescents (all age) preschool children (ages 2–5 years), school‐going children (ages of above 5 years) and adolescents till 18	Fortification is potentially an effective strategy but evidence from the developing world is scarce	The techniques of fortification are not mentioned	Das et al. (2013)
Wheat flour	Iron, vitamin A,	Fortification level for wheat flour is as follows: iron (as NaFeEDTA) 5 ppm, iron (as Electrolytic iron) 50 ppm, folic acid (as folic acid) 1.3 ppm, vitamin B12 (as cyanocobalamin) 0.01 ppm, and vitamin A (as vitamin A palmitate) 1.5 ppm	Children aged 6–59 month and women	—	—	Bhagwat et al. (2014)
Soybean oil	Iron, vitamin D	Soybean oil fortification level is as follows: vitamin A (as retinyl palmitate) 25,000 IU/kg of oil; vitamin D2 2,000 IU/kg of oil	Children aged 6–59 months Women	—	—	Bhagwat et al. (2014)
Milk	Iron, vitamin D	Milk Fortification level is: Vitamin A (as Retinyl acetate, water miscible) 2,000 IU/L of milk, Vitamin D−2,400 IU/L of milk	Children aged 6–59 months ‐Women	—	—	Bhagwat et al. (2014)

### 3.3 Historical trends and impacts of food fortification

For fixing the iodine deficiency (cause of goiter) that happened early in the 20th century, public health opted for the first time to food fortification (Backstrand, Allen, Black, de Mata, & Pelto, 2002; Mannar & Hurrell, 2018). Indeed, the first fortified food was the iodized salt to prevent goiter (Mannar & Hurrell, 2018) that was introduced in Switzerland and Michigan (United States) in 1923 and 1924, respectively (Abdullahi, Zainab, Pedavoah, Sumayya, & Ibrahim, 2014; Mannar & Hurrell, 2018; Marine & Kimball, 1920). In the same period, many vitamins were isolated and their molecular structures elucidated. As a result, it was possible to produce vitamins for fortifying foods at a large scale. In the 1930s, iron was mainly used to fortify cereal flours and products for a large population such as fish sauce (Vietnam), soy sauce (China), and rice (Philippines; Mannar & Gallego, 2002). Since 1938, niacin had been added to bread in the United States. From the early 1940s onwards, fortification of cereal products with thiamine, riboflavin, and niacin (Kyritsi, Tzia, & Karathanos, 2011) became a common practice. Meanwhile, rice fortification received considerable attention due to the great importance of rice in children's nutrition. Margarine was fortified with vitamin A (FAO & OMS, 2006) in Denmark and milk with vitamin D in the United States (Laforest et al., 2007). The fortification of sugar with vitamin A has been introduced for the first time during the 1970s in Guatemala, followed by other Costa Rica, Honduras, and El Salvador, for reaching up to 80% (Honduras) and 95% (Guatemala and El Salvador) of households (Mora, Dary, Chinchilla, & Arroyave, 2000). The success of this fortification allowed many countries to effectively combat micronutrient deficiencies in populations. Enriching flour and cereal products moved from the use of iron, niacin, riboflavin, and thiamin to the use of folic acid in 1996 (Food and Drug Administration, United States) for enriching breads, flours, corn meals, and rice in order to address neural tube defects in newborns (Backstrand et al., 2002). Therefore, folic acid fortification of wheat became widespread, a strategy adopted by Canada and the United States and about 20 Latin American countries (Samaniego‐Vaesken, Alonso‐Aperte, & Varela‐Moreiras, 2010). Thus, folic acid was added to flour on a mandatory basis in over 60 countries to prevent neural tube birth defects (Liyanage & Zlotkin, 2002; Oakley & Tulchinsky, 2010).

Other food vehicles fortified with vitamin A, besides sugar, include fats and oils, tea, cereals, flour, monosodium glutamate, and instant noodles, as well as milk or milk powder, whole wheat, rice, salt, soybean oil, and infant formulas (Lotfi, Venkatesh Mannar, Merx, & Heuvel, 1996). In Asia, the red palm oil was used as a vitamin A fortificant added to other edible oils (Solomons, 1998). Currently, fortifying foods with vitamin A are common in 29 developing countries (Mason et al., 2014).

The huge success of salt iodization was likely a critical factor in generating support for other fortification initiatives. In Ghana, food fortification began in 1996 when legislation was passed to enforce salt iodization. Salt iodization has been ongoing with the target of covering at least 90% of the population (Nyumuah et al., 2012). Through Africa, maize meal and bread were shown to be the most commonly consumed staples; hence, vitamin A, iron, zinc, folic acid, thiamin, niacin, vitamin B, and riboflavin have been added to maize meal and wheat flour with the aim of improving the growth and micronutrient status of undernourished children (Steyn, Nel, & Labadarios, 2008).

### 3.4 Global challenges of food fortification

#### 3.4.1 Socioeconomic factors hindering the practice of food fortification

In most developing countries, national policies do not provide the appropriate importance to food fortification. Due to the low development of their industry sector (including food‐processing industries), it is hard to reach poor people who really need fortified foods. Increased food prices remain an issue in undermining food security and livelihoods of the poor. Despite various international aids, expensive foods are still not accessible because unaffordable for vulnerable groups who often grow and process their own staple foods. Temple and Steyn (2011) demonstrated that purchasing healthier food items resulted in 69% higher daily costs. For food fortification development, the absence of centralized food‐processing units is a limiting factor. Another one is the lack of simple and affordable technology that can use stable and bioavailable nutrients while maintaining the commonly preferred taste and appearance of foods. The major challenges of developing countries regarding food fortification rely on the lack of industrial concentration and the socioeconomical level of the large segments of the population that does not allow them affording expensive foods (Bhagwat et al., 2014). Major challenges to local‐scale fortification programs include the initial cost of the mixing equipment, the price of the premix, achieving and maintaining an adequate standard of quality control, and sustaining monitoring and distribution systems.

#### 3.4.2 Technical limits to the practice of food fortification

Technical fortification challenges rely on (a) nonappropriateness of fortification causing nutrients' loss, (b) sunlight exposure of fortified foods by retailers, (c) nonregular monitoring and unreliable quality control procedures by companies. The most important challenge is to ensure a regulatory monitoring that aims at meeting fortified foods to national fortification standards (Method & Tulchinsky, 2015). Governments in developing countries may not have the resources to effectively monitor compliance, especially when there are many small processing companies operating. As Luthringer, Rowe, Vossenaar, and Garrett (2015) showed, financial inputs for monitoring have a proportional significant effect on the effectiveness of detection and enforcement of noncompliant and under fortified products. Cooperative working relationships between regulatory agencies and food producers will be a useful strategy for successful fortification programs. Challenges such as choosing appropriate fortification vehicles, reaching target populations, avoiding overconsumption in nontarget groups, and monitoring nutritional status are relevant to all countries because they occur everywhere where there is an attempt to fortify foods to optimize intake and nutritional status (Dwyer et al., 2015). In Sub‐Saharan Africa, dietary diversification can be used effectively to enrich indigenous and traditional foods.

#### 3.4.3 Communication factors limiting the practice of food fortification

Apart from the socioeconomical and technical challenges, efforts must be done to inform consumers about the existence and importance of fortified foods for their well (Pambo, Otieno, & Okello, 2014). Indeed, media are the most important source of nutrition information and fortification awareness. However, reliable information about food fortification is not widely available for instance some African countries. Under those circumstances, nonfortified food has a price advantage because of the absence of a mandatory provision and low levels of awareness on the benefits of fortification.

### 3.5 From classical food fortification to food‐to‐food fortification

Recently, food fortification has motivated many efforts worldwide but it still faces several issues in developing countries. The major challenge of classical food fortification relies on the whole food processing from production to consumption in developing countries, in addition to economic factors especially in Sub‐Saharan Africa. Indeed, successful food fortification has a complex relationship with the level of economic development. For achieving a good food fortification, efforts should be done in food processing (by using modern techniques); quality control and monitoring systems; transport (reliable distribution infrastructure); regulatory support; and management of market dynamics (in a way to make ease access to food for low‐income families). The unbalanced accessibility to staple foods is a major limit for poor populations who are actually the ones at the highest risk of micronutrient deficiency, to benefit of fortified foods (Wimalawansa, 2013). It is then important to find a technique that uses the fortificant with a highly accessible (i.e., financially and physically) vehicle, already used by the target population. More and more, the aim of food fortification is to improve people health instead of deficiencies' prevention (Dwyer et al., 2015). Thus, to overcome all these challenges, development of nutritious and cheap foods from locally available foods is important. Efforts are being made by employing food‐to‐food fortification (Onuoha & Ene‐Obong, 2005). Food‐to‐food fortification is an approach that uses an interesting (contain useful amounts of micronutrients), available, and accessible local resource (plant or animal) to fortify another food (Uvere, Onyekwere, & Ngoddy, 2010). Though, it is difficult to find an interesting resource that meets the availability and affordable accessibility conditions. It is also necessary that the fortificant food may not affect sensory properties of the food that needs to be fortified. In most cases, it is preferable to use food vehicles that are centrally processed, and to have the support of the food industry for an effective impact of food‐to‐food fortification. For this technique, the rate of fortification varies considerably (1%–50%) and depends on the compatibility of the vehicle (the staple food) and the fortificant. For both classical food fortification and food‐to‐food fortification, the main objective is to improve the nutritional quality of the fortified food without losing sight of the acceptability criteria (mainly the food organoleptic quality).

### 3.6 Practices and benefits of food‐to‐food fortification

#### 3.6.1 Practices and benefits

Food‐to‐food fortification often uses foods that are available in the area of the target population to enhance nutrient intake. This approach consists of selecting and associating foods (a common staple and a fortifying food) in such a way to optimize the bioavailability of interesting micronutrients to consumers. For example, in Nigeria, *ogi,* a fermented cereal‐based dough produced mostly from maize, is fortified with baobab fruit powder (rich in vitamins A, C, E, and F; proteins; fiber; carbohydrates; iron; zinc; calcium; and potassium; Adejuyitan, Abioye, Otunola, & Oyewole, 2012), and *tapioca* made from cassava tubers is fortified with soybean flour (carbohydrates, fiber; Kolapo & Sanni, 2015).

Worldwide, food‐to‐food fortification was studied for fighting against malnutrition (Abdullahi et al., 2014; Abioye & Aka, 2015; Adejuyitan et al., 2012; Adenuga, 2010; Ajanaku, Ogunniran, Ajani, James, & Nwinyi, 2010; De Brito, Garruti, & Silva, 2007; Giwa & Ibrahim, 2012; Kolapo & Sanni, 2015; Lelana, Purnomosari, & Husni, 2003; Meite, Kouame, Amanii, Katii‐Coulibaly, & Offoumou, 2008; Nadeem, Javid, Abdullah, Arif, & Mahmood, 2012; Okafor, Okafor, Ozumba, & Elemo, 2012; Oluwamukomi & Jolayemi, 2012; Onuoha & Ene‐Obong, 2005; Salem, Salama, Hassanein, & El Ghandour, 2013; Samuel, Otegbayo, & Alalade, 2012). In Benin, it is common to see the association of local food resources, such as leaves (moringa), fruit (pawpaw, mango, *plantain*), seeds (from watermelon), legumes (soybean), and even edible mushrooms, as fortifying food to improve nutrient intake of some deficient foods (*gari*, *tapioca*, and wheat flour bread). The main role of a fortifying food is to fill nutritional, sensory, biological, and physical gaps. Food‐to‐food fortification usually provides energy, proteins, fat, fiber, carbohydrates, phosphorus, iron, zinc, potassium, manganese, sodium, calcium, and vitamin C.

Table 2 summarizes the evidence on the effectiveness of food‐to‐food fortification interventions. Examples of food‐to‐food fortification are also presented by Vuong (2000) for traditional rice dishes in Vietnam, liver chips as a snack in southern Thailand (Wasantwisut, Chittchang, & Sinawat, 2000), and red palm oil incorporated into biscuits in child‐feeding programs in South Africa (van Stuijvenberg & Benadé, 2000). In Northeast Brazil, the pulp from the buriti fruit (*Mauritia vinifera* Mart.) is used daily as a dietary supplement to children at high risk (12 g containing ~800 µg β‐carotene or 134 µg retinol) to resolve or attenuate clinical signs of vitamin A deficiency (Mariath, Lima, & Santos, 1989). In South India, the β‐carotene‐rich blue‐green alga Spirulina, prepared as a sweetened product suitable as a snack, improved vitamin A status of preschoolers attending daycare centers (Annapuma, Deosthale, & Bamji, 1999). Odinakachukwu, Nwosu, Ngozi, Ngozi, and Aloysius (2014) revealed that *Moringa oleifera* fortification of infant complementary food improved its nutritional quality. Incorporation of pulverized *M. oleifera* leaves in infant foods could diversify food intake and ensure food and nutrition security. An assessment of iron absorption was conducted by Cercamondi et al. (2014) in Burkina Faso and indicated any change in the iron quantity absorbed by young women when they ate a meal constituted of a maize paste accompanied by iron‐improved leaf‐based and traditional amaranth sauce. Therefore, increasing leafy vegetables in a meal could not be enough to provide additional bioavailable iron. Steyn et al. (2008) examined dietary intake of children at population level who consume fortified staple foods in South Africa. Results indicate that the addition of micronutrients to staple foods made a significant difference to the intake of vitamin A, thiamine, niacin, vitamin B, folic acid, and iron. These improvements were particularly important in rural areas where children had the lowest mean dietary micronutrient intake.

**Table 2:** Examples on effectiveness of food‐to‐food fortification

Food used for fortification (fortificant)	Vehicles (basic food)	Fortifier	Technique of fortification	Advantages	Limits	References
Flour of *Citrullus lanatus* seeds	Bread from wheat flour	Proteins, fat, and ash	Wheat flour is substituted for 5%, 15%, and 20% by defatted seeds of *C. lanatus*. The substitution rate until 20% of the wheat flour by the seeds of *C. lanatus* was acceptable in terms of sensorial and physical properties with improvement of nutritional qualities		The carbohydrate content in fortified bread is lower than the one in bread made by 100% of wheat flour	Meite et al. (2008)
Soybean (*Glycine max*) and melon seed (*Citrullus vulgaris*): soybean–melon protein supplements	“Gari,” a fermented and toasted cassava granule	Protein	“Gari,” a fermented and toasted cassava granule, was enriched with 10% of full fat soy–melon protein supplements, at different processing stages (after toasting and before toasting)	The gari enriched prior to toasting was better in most of the pasting properties, bulk density, and gel strength	The enriched “gari” sample exhibited high setback and breakdown viscosity values of indicating that its paste will have lower stability against retrogradation than the un‐enriched gari samples	Oluwamukomi and Jolayemi (2012)
Java tilapia flour (Fish)	Plain cracker	Protein	Plain cracker was fortified with varying proportions of Java tilapia flour 5%, 10%, 15%, and 20%	Fortification of plain cracker with Java tilapia flour increase protein content of plain cracker. Fortification of cracker with 5% of Java tilapia is acceptable	Fortification of Plain cracker with Java tilapia flour decreases relative volumetric expansion and sensory properties of the cracker	Lelana et al. (2003)
Soybean	Gari from the tubers of cassava (*Manihot esculenta*)	Crude proteins, phosphorus, fat, and ash, manganese, iron, copper, zinc, and potassium	Gari was fortified with soybean flour or soybean residue at 25% of dry weight	Soybean flour increased the macronutrient and micronutrient content of the fortified gari	Difficulty in processing soybean residue‐fortified products	Kolapo and Sanni (2015)
	Tapioca from cassava tubers (*Manihot esculenta*)	Crude protein, phosphorus, fat, and ash, manganese, iron, copper, zinc, and potassium	Tapioca was fortified with soybean flour or soybean residue at 25% of dry weight	Soybean flour increased the macronutrient and micronutrient content of the fortified tapioca	Difficulty in processing soybean residue‐fortified products	Kolapo and Sanni (2015)
Acerola (*Malpighia emarginata*), mango fruit pulps, or soy extract	Tapioca	Vitamin C and protein	The products were formulated with dried tapioca starch (63%) supplemented by acerola or mango fruit pulps or by soy extract in selected combinations (37%)	This study indicated that the developed tapioca products had good sensory acceptance with increase in nutritional value	—	De Brito et al. (2007)
Soybean flour (full fat)	Tapioca	Crude protein, crude fat, crude fiber, ash, energy, sodium, potassium, calcium, and phosphorus	Tapioca was enriched with varying proportions of soybean flour (0, 85%–15%, 75%–25%, 50%–50%) to produce soy‐tapioca	Soy fortification resulted in improvement of the nutrient composition in terms of protein, fat, energy, and mineral contents. Soy enhanced tapioca samples had a low level of antinutritional components, making them safe for consumption. There was a decrease in the cyanogenic potential and an increase in the level of trypsin inhibitor as soy‐substitution increased	—	Samuel et al. (2012)
Pawpaw fruit slurry	Sorghum‐ogi	Protein, ash, fat, vitamins c, and sugar	A 100 g of ogi (dry basis) was mixed with 0, 20, 40, 60, 80, and 100 g of papaw slurries (dry basis)	Blends with 40% pawpaw addition and beyond were acceptable in improving the nutritive value of ogi	There was no apparent effect in pawpaw addition on the pH and titrable acidity of ogi	Ajanaku et al. (2010)
Cowpea and peanut	Sweet potato‐based infant weaning food	Protein, ash, fat, crude fiber, and carbohydrates	The flours were combined in specific ratios (sweet potato: 60%, 65%, and 70%; cowpea: 25%, and 15%, 15%; and peanut 15%, 25%, and 15%)	Fortification 60% sweet potato, and less than 25% cowpea and 15% peanut flour was acceptable in terms of sensory property with increase in nutritional values.	Infant weaning food developed using sweet potato, cowpea, and peanut showed a decrease in the sensory quality of the weaning food	Adenuga (2010)
Defatted Soy Flour	Tapioca meal	Protein and ash	The cassava starch and defatted soy flour were mixed in the ratio 100:0, 95:5, 90:10, 85:15, and 80:20 to produce tapioca meal	The sample with 80:20 cassava starch and defatted soy flour had the highest protein content and the least moisture content. It was also rated highest in terms of overall acceptability from the sensory evaluation	—	Balogun, Karim, Kolawole, & Solarin (2012)
Mushroom flour and spices (*Alllium sativum*)	Cookies from wheat flour	—	Wheat flour was used to substitute mushroom flour at the ratio of 70:30, 50:50, 30:70 and with spices (*A. sativum*) which concentration range from 5, 10, and 15 g, respectively	Produce a significant effect on the physical properties as the diameter, thickness, and spread factor varies significantly at the probability level less than 0.05 as the concentration of spice (*A. sativum*) increases.	—	Giwa and Ibrahim (2012)
Oyster Mushroom (*Pleurotus plumonarius*) Powder	Bread from wheat flour	Crude Proteins, ash, and crude fiber	Bread containing graded levels of mushroom powder were produced by replacement of wheat flour with 0, 5%, 10%, 15%, 20%, and 25% mushroom powder	The substitution rate of 15% of the wheat flour by the mushroom powder was acceptable in terms of, sensorial and physical properties with increase in nutritional value	The acceptability decreased with increase in inclusion of mushroom powder. Bread with 25% mushroom powder was the least acceptable	Okafor et al. (2012)
Baobab (*Adansonia digitata*) fruit Pulp powder	Yoghurt from milk	lipid, fiber, protein, and mineral	*A. digitata* fruit Pulp powder was used to fortified yoghurt production in ratios of 4:1, 3:2, 2:3, 1:4, and 5:0. Incorporation of *A. digitata* fruit pulp increased the bioavailability of nutrients, minerals, and a volatile metabolite with medicinal properties.	Increase in nutritional value	—	Abdullahi et al. (2014)
African locust bean (*Parkia biglobosa*) pulp	Functional bread products from wheat flour	Protein, fat, ash, and fiber	The functional bread produced from wheat flour was fortified with 0, 5%, 10%, 15%, 20%, 30%, and 40% of Parkia flour	The investigation shows that there was significant improvement in the bread‐resistant starch content and nutritional quality on addition of Parkia flour. The sensory evaluation also indicated that 5%, 10%, and 15% Parkia flour bread was the most acceptable bread.	Bread with 40% Parkia flour addition had significantly poor appearance, texture, and pronounced Parkia taste and aroma	Sankhon et al. (2013)
Baobab fruit pulp	“Ogi” powder produced from maize	Minerals and vitamin particularly vitamin C	“Ogi” produced from maize was fortified with baobab fruit powder at substitution levels of 0, 10, 20, and 50%.	All the fortified samples were acceptable. Ogi supplemented with baobab fruit powder had higher minerals and vitamin contents particularly vitamin C	Decrease of protein, crude fiber, fat, and carbohydrate	Adejuyitan et al. (2012)
*Moringa oleifera leaf powder*	Buttermilk	The protein, ash, total solids, calcium, iron, and vitamins of B‐group	Dry leaves of *Moringa oleifera* were incorporated into buttermilk at three different concentrations 1%, 2%, and 3%	Dry leaves of *Moringa oleifera* can be used at T3 level (3% addition) to formulate fortified buttermilk with increased nutritional value and acceptable sensory attributes.	The low color score was observed	Nadeem et al. (2012)
Dry leaves of *Moringa oleifera*	Labneh cheese	Ca, Fe, Zn and Si, vitamins A, B1, B2, and E	Dry leaves of *M. oleifera* were added to Labneh cheese at concentration 1%, 2%, or 3%.	Labneh fortified with dry leaves of *M. oleifera* characterized with high biological value (BV), true protein digestibility (TD), and net protein utilization (NPU) and acceptable up to 2%.	The addition of dry leaves of *M. oleifera* tended to make the color (appearance) greener	Salem et al. (2013)
Moringa leaf powder	Maize‐ogi	Protein, fat, ash, crude fiber, calcium, magnesium, iron, potassium, zinc, and copper	The “ogi” produced from maize was fortified with moringa leaves at substitution levels of 0, 10%, and 15%.	The ogi sample with 10% moringa leaves substitution was rated close to the unfortified ogi sample. Improvement of the nutritional and sensory qualities	The swelling capacity decreased with increase in the level of moringa leaves substitution	Abioye and Aka (2015)

#### 3.6.2 Fortification/substitution rate

The adequate rate of fortification varies considerably between 1% and 50% according to the compatibility between the fortifying and the vehicle foods. For instance, 2% and 3% of dry leaves of *M. oleifera* can be enough, despite the low rate of fortification, to enrich the nutritional properties of *Labneh* cheese (Abdullahi et al., 2014) and buttermilk, respectively. As well as the fortification rate is important, for example, 15% of dry *M. oleifera* leaves in maize‐*ogi* (Abioye & Aka, 2015), the physical properties of the fortificant have to be analyzed in order to select the rate that will provide an optimized nutrient intake and will maintain the food acceptability by consumers. As such, next to the laboratory formulation of the fortified food, a sensorial evaluation is necessary to assess acceptability levels.

However, for *ogi* produced from sorghum and fortified with pawpaw fruit at substitution levels of 0, 20%, 40%, and 60%, blends with 40% pawpaw and more were acceptable for improving the nutritional value of *ogi* without affecting the sensory quality (Ajanaku et al., 2010). According to Sankhon, Amadou, and Yao (2013), sensory evaluation indicated that 5%, 10%, and 15% of parkia‐fortified flour bread were the most acceptable breads. A substitution rate of 15% of wheat flour by mushroom powder to produce bread was also acceptable in terms of sensorial and physical properties (Okafor et al., 2012).

Substitution of 20% of wheat flour by the seeds of watermelon (*Citrullus lanatus*) was acceptable in terms of sensorial and physical properties but the carbohydrate content in the fortified bread was lower than in bread consisting for 100% of wheat flour. Fortification of wheat flour with *M. oleifera* seed flour in bread production for up to 15% reportedly increased protein content by approximately 67% without significantly altering the sensory properties (Ogunsina, Radha, & Govardhan Singh, 2010). *Ogi* based on maize fortified with baobab fruit powder was found to be acceptable for up to 50% substitution with also a decrease in protein, crude fiber, fat, and carbohydrates. Apparently, fortification could cause a reduction in the levels of certain nutrients in the basic food (vehicle) while improving others.

#### 3.6.3 Step by step toward food‐to‐food fortification

For a successful fortification, the aim and the approach must be clearly defined before beginning the process. Obviously, the first thing to do is the identification of nutrients that lack and need to be provided. Then, the most appropriate fortificant has to be identified, considering the food habits of the target population and the most appropriate techniques for fortification. According to FAO and OMS (2006), a vehicle food may meet four main features: (a) Its consumption must be common to a large population including the most vulnerable one, (b) its consumption must be regular and in consistent quantity, (c) it should be centrally processed, and (d) it must allow a nutrient premix to be added relatively easily using low‐cost technology, and in such a way so as to ensure an even distribution within batches of the product.

For optimizing the acceptability of the fortified food and preventing undesirable chemical reactions (Maillard reaction) (Adenuga, 2010) when using the food‐to‐food approach, some agents can be added to improve its sensory properties (Lelana et al., 2003). The step at which the fortificant is added to the vehicle also impacts the physical properties of the fortified food. For example, in the case of *gari* fortified with soybean flour, the soybean flour is added to the *gari* before or after toasting (Oluwamukomi & Jolayemi, 2012). Fortificant may also be added to some fermented food vehicle before or after fermentation.

### 3.7 Way forward

Food fortification is necessary for both developed and developing countries to ensure optimal levels of essential nutrients in processed foods, improving their suitability for human nutrition and preventing many diseases. To ensure the success and sustainability of food fortification, programs should be implemented in concert with cultural consideration; poverty reduction programs; and other agricultural, health, education, and social intervention programs that promote the consumption and utilization of adequate quantities of good quality nutritious foods. According to Mildon, Klaas, O'Leary, and Yiannakis (2015), further work is needed to determine contextually feasible and sustainable mechanisms for premix supply, quality control, and cost recovery. Incorporating this work into national fortification frameworks is recommended for countries where a significant proportion of the population may have limited access to commercially fortified foods and appropriate legislation is needed to overcome challenges faced by classical food fortification (WHO, 2000). Food fortification should thus be viewed as a complementary strategy for improving micronutrient status of the population. In many countries, basic information on existing dietary intake is lacking. Accurately assessing intake of fortification vehicles is needed to determine the dietary impact of any fortification program (Dwyer et al., 2015).

Thus, continuous production and monitoring of quality fortified foods are essential. More emphasis is needed to determine locally sustainable mechanisms for quality control and cost recovery of food fortification in Africa. Furthermore, the foods to fortify as well as the micronutrient mix or food fortificant must be chosen carefully, to identify the most appropriate vehicles for food fortification as well as target the population at risk of inadequacy without creating excessive intake for other subgroups of the population. Fortification must be applied thoughtfully, its effects monitored diligently, and the public informed effectively about its role in dietary intake through appropriate labeling and other sources of consumer education. For research purposes, databases must be constantly updated to reflect the rapidly evolving marketplace, so that the contribution of both added and intrinsic micronutrients accurately estimates population intakes.

## 4 CONCLUSION

The best way to prevent micronutrient malnutrition is to ensure consumption of a balanced diet that is adequate in every nutrient. Studies on fortification of foods have shown promising results in the control and prevention of micronutrient deficiency among vulnerable populations, especially women and children. Food fortification is necessary for developed and developing countries to ensure essential nutrients in processed foods, improving their suitability for human nutrition. However, fortification, though promising, is not the only answer to the global widespread nutritional deficiencies. A mix of many food‐based approaches is needed to tackle undernutrition, especially in developing countries.

## CONFLICT OF INTEREST

The authors declare that they have no conflict of interest.

## ETHICAL REVIEW

This study does not involve any human or animal testing.

## ENDNOTE

1 Food‐to‐food fortification refers to the addition of a given food to other processed foods in a way that reinforces them.
